# Supplementary material for: The Genotype-Phenotype Correlation in Human 5α-Reductase Type 2 Deficiency: Classified and Analyzed from a SRD5A2 Structural Perspective
Source: Int J Mol Sci. 2023 Feb 7;24(4):3297. doi: 10.3390/ijms24043297 (PMC9966410; doi:10.3390/ijms24043297)
Supplement: Supplementary file 1 [file ijms-24-03297-s001.zip › ijms-2202006-supplementary.pdf]

Figure S1. The *SRD5A2* mutational spectrum and frequencies in 19 Korean children with 5αRD2

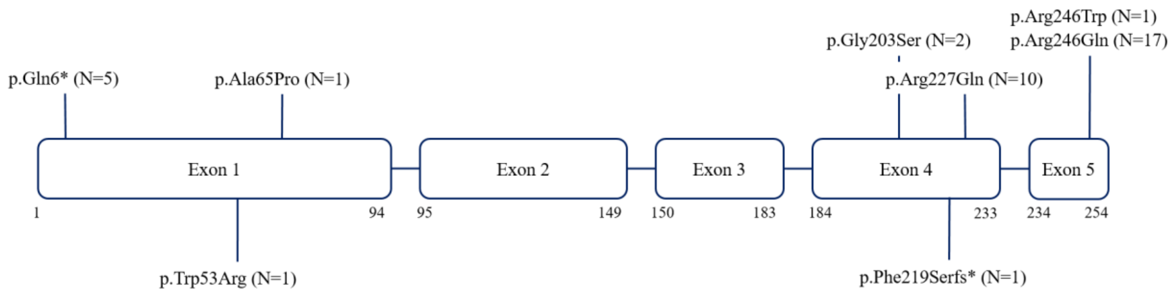

Table S1. The testosterone (T), dihydrotestosterone (DHT), and T/DHT ratio levels at baseline and after human chorionic gonadotropin (hCG) stimulation

	Patient No.	Age (yr)	Baseline			After hCG stimulation		
			T (ng/dL)	DHT (ng/dL)	T/DHT	T (ng/dL)	DHT (ng/dL)	T/DHT
p.R227Q	1	9.7	<2.5	2	<1.3	106.3	8	13.3
		13.1	224.6	12	18.7			
		13.3	121.3	7	17.3			
		16.0	483.0	10	48.3			
		16.5	468.0	9	52.0			
		17.0	425.0	14	30.4			
	2	1.3	<2.5	5	<0.5	507.0	9	56.3
	3	0.5	10	2	5.0	217.6	22	9.9
	4	0.9	<2.5	-	-	309.0	9	34.3
	5	0.8	<2.5	2	<1.3	610.0	35	17.4
	6	5.5	<2.5	2	<1.3	300.9	7	43.0
	7	12.7	559.3	9	62.1	1149.0	18	63.8
	8	11.8	17.3	5	3.5	203.7	8	25.5
		13.5	158	11	14.4			
		13.9	182.3	14	13.0			
		17.9	266.0	10	26.6			
	9	0.6	<2.5	2	<1.3	427.1	25	17.1
Non-p.R227Q	10	0.0	192.0	44	4.4	587.0	69	8.5
	11	0.7	<2.5	2	<1.3	846.8	24	35.3
	12	0.0	154.0	22	7.0	Not done		
	13	4.6	<2.5	2	<1.3	166.4	7	23.8
	14	16.5	623.3	48	13.0	1267.0	83	15.3
	15	0.1	118.7	23	5.2	458.2	65	7.0
		12.4	170.5	23	7.4	660.7	44	15.0
	16	0.1	240.8	24	10.0	567.3	46	12.3
	17	2.6	4.0	1	4.0	279.7	3	93.2
	18	10.5	7.4	4	1.9	37.0	4	9.3
	19	9.9	<2.5	5	<0.5	330.0	5	66.0
	Median	9.8	120.0	9.0	5.2	427.1	18.0	17.4
	Minimum	0.0	<2.5	1.0	<0.5	37.0	3.0	7.0
	Maximum	17.9	623.3	48.0	62.1	1267.0	83.0	93.2

Table S2. The testosterone (T), dihydrotestosterone (DHT), and T/DHT ratio levels at baseline and after human chorionic gonadotropin (hCG) stimulation according to age

Age	N	Baseline			N	After hCG stimulation		
		T (ng/dL)	DHT (ng/dL)	T/DHT		T (ng/dL)	DHT (ng/dL)	T/DHT
< 6 mon	4*	173.0 (118.7–240.8)	23.5 (22.0–44.0)	6.1 (4.4–10.0)	3*	567.3 (458.2–587.0)	65.0 (46.0–69.0)	8.5 (7.0–12.3)
6 mon–12 yr	12	2.5 (2.5–17.3)	2.0 (1.0–5.0)	1.3 (0.5–5.0)	13	300.9 (37.0–846.8.0)	8.0 (3.0–35.0)	25.5 (9.3–93.2)
p.R227Q	7	2.5 (2.5–17.3)	2.0 (2.0–5.0)	1.3 (0.5–5.0)	8	305.0 (106.3–610.0)	9.0 (7.0–35.0)	21.4 (9.9–56.3)
Non-p.R227Q	5	2.5 (2.5–7.4)	2.0 (1.0–5.0)	1.3 (0.5–4.0)	5	279.7 (37.0–846.8)	5.0 (3.0–24.0)	35.3 (9.3–93.2)
	P				*P*			
> 12 yr	11†	266.0 (121.3–623.3)	11.0 (7.0–48.0)	18.7 (7.4–62.1)	3	1149.0 (660.7–1267.0)	44.0 (18.0–83.0)	15.3 (15.0–63.8)

All data shows as median (min–max).

*all participants are non-p.R227Q variant allele group.

†Only two participants are non-p.R227Q variant group.

Note S1. List of 404 genes included in the custom panel for DSD-related genes

ABCA1, ABCC8, ABCG1, ABCG5, ABCG8, ADRB2, ADRB3, AGL, AGPAT2, AGRP, AIP, AIRE, AKAP2, AKR1C2, AKR1C4, AKT2, ALMS1, AMH, AMHR2, ANGPTL3, ANOS1, AP2S1, APC, APOA1, APOA4, APOA5, APOB, APOC2, APOC3, APOE, APPL1, AQP2, AR, ARAP1, ARL4D, ARL6, ARMC5, ARX, ATP1A1, ATP2B3, ATP7A, ATRX, AVP, AVPR2, BBS1, BBS10, BBS12, BBS2, BBS4, BBS5, BBS7, BBS9, BDNF, BLK, BMP15, BRAF, BSCL2, BTK, CA2, CACNA1D, CARTPT, CASR, CAV1, CAV2, CBX2, CCND1, CDC73, CDKL5, CDKN1A, CDKN1B, CDKN1C, CDKN2B, CDKN2C, CEL, CEP290, CEP41, CETP, CHD7, CIDEC, CISD2, CLCN7, COL1A1, COL1A2, COL3A1, COL5A1, COL5A2, COL9A1, COL9A2, COL9A3, COLEC11, COMP, CP, CREB3L3, CREBBP, CRTAP, CRTC1, CRY2, CUL3, CUL4B, CYB5A, CYP11A1, CYP11B1, CYP11B2, CYP17A1, CYP19A1, CYP21A2, DDH2, DHCR24, DHCR7, DHH, DMRT1, DMRT2, DNAJC3, DUSP6, DYRK1B, EGLN1, EIF2AK3, ELN, EMX2, ENPP1, EPAS1, FBP1, FERMT3, FEZF1, FGD1, FGF17, FGF8, FGF9, FGFR1, FGFR2, FH, FIGLA, FKBP10, FLRT3, FOXC2, FOXE1, FOXL2, FOXP3, FRAS1, FREM2, FSHB, FSHR, G6PC, GALNT2, GATA3, GATA4, GATA6, GBE1, GCK, GCKR, GCM2, GDF9, GHR, GHRL, GLI2, GLI3, GLIS3, GLUD1, GNA11, GNAS, GNRH1, GNRHR, GPD1, GPIHBP1, GPR161, GRIP1, GYS2, HADH, HADHA, HADHB, HESX1, HFE, HHIP, HIRA, HNF1A, HNF1B, HNF4A, HOXA13, HRAS, HS6ST1, HSD17B3, HSD3B1, HSD3B2, ICK, IER3IP1, IFIH1, IFITM5, IGFALS, IKBKG, IL17RD, INS, INSL3, INSR, IRF6, IYD, KAT6B, KCNJ11, KCNJ5, KIF1B, KISS1, KISS1R, KLF11, KLHL3, KRAS, LCAT, LDLR, LDLRAP1, LEP, LEPR, LHB, LHCGR, LHX3, LHX4, LIMK1, LIPA, LIPC, LIPE, LIPG, LMF1, LMNA, LMNB2, LPIN1, LPL, LRP5, LRP6, MADD, MAGEL2, MAMLD1, MAP2K1, MAP2K2, MAP3K1, MATN3, MAX, MC2R, MC3R, MC4R, MCM4, MEF2A, MEN1, MKKS, MKS1, MLXIPL, MTTP, MYLIP, NEUROD1, NEUROG3, NF1, NIPBL, NKX2-1, NKX2-5, NLGN4X, NNT, NOBOX, NOTCH2, NPC1L1, NR0B1, NR0B2, NR5A1, NRAS, NSMF, NTRK1, NTRK2, OCRL, OPHN1, OSTM1, OTC, OTX1, OTX2, P3H1, PAX4, PAX8, PAX9, PCK1, PCK2, PCSK1, PCSK9, PDX1, PHF6, PHIP, PHKA2, PHKB, PHKG2, PITX2, PLEKHM1, PLIN1, PLOD1, PLOD2, PLTP, PNPLA2, POLD1, POMC, POR, POU1F1, PPARA, PPARG, PPARGC1B, PPIB, PRKAR1A, PROK2, PROKR2, PROP1, PSMC3IP, PTEN, PTF1A, PTH, PTH1R, PTPN11, PTRF, PYGL, PYY, RASGRP2, RB1, RET, RFX6, ROR2, RSPO1, RXFP2, SALL1, SAR1B, SCARB1, SDC3, SDCCAG8, SDHA, SDHAF2, SDHB, SDHC, SDHD, SECISBP2, SEMA3A, SEMA3E, SERPINF1, SERPINH1, SIM1, SLC16A1, SLC16A2, SLC26A2, SLC26A4, SLC2A2, SLC37A4, SLC39A13, SLC5A5, SLC9A3R1, SORT1, SOX10, SOX2, SOX3, SOX9, SP7, SPECC1L, SPRY4, SRA1, SRD5A2, SREBF1, SRXX2, SRXX3, SRXY10, SRY, STAP1, STAR, STK11, STS, STX16, SULT2A1, TAC3, TACR3, TBC1D4, TBCE, TCIRG1, TCTN3, TG, THRA, THRB, TMEM127, TMEM38B, TNFRSF11A, TNFSF11, TP53, TPO, TRHR, TRIB1, TRIM32, TSHB, TSHR, TSPYL1, TTC8, UBR1, UCP1, UCP2, UCP3, VHL, VPS13B, WDPCP, WDR11, WFS1, WNK1, WNK4, WNT1, WNT4, WNT7A, WRN, WT1, WWOX, ZFP57, ZFPM2
